# S7-4 Active transport to physical exercise places among older adults living in different urban zones

**DOI:** 10.1093/eurpub/ckad133.036

**Published:** 2023-09-11

**Authors:** Kirsi E Keskinen, Essi-Mari Tuomola, Taina Rantanen, Erja Portegijs

**Affiliations:** Faculty of Sport and Health Sciences and Gerontology Research Center, University of Jyväskylä, Jyväskylä, Finland; School of Resource Wisdom, University of Jyväskylä, Jyväskylä, Finland; Faculty of Sport and Health Sciences and Gerontology Research Center, University of Jyväskylä, Jyväskylä, Finland; Faculty of Sport and Health Sciences and Gerontology Research Center, University of Jyväskylä, Jyväskylä, Finland; Center for Human Movement Sciences, University Medical Center Groningen, University of Groningen, Groningen, The Netherlands

## Abstract

**Purpose:**

Motorized transport to and from places of physical exercise (PE) causes considerable amounts of carbon emissions. We study how locations of home and PE place on urban zones (UZs), reflecting options for transport modes, relate to use of active transport (AT) among older adults. No previous knowledge on the topic exists.

**Methods:**

Data of AGNES study participants reporting at least one regular PE place <10 km from home (n = 819, mean 79 years, 58% women, Jyväskylä Finland) were combined with geospatial data on UZ. Using digital mapping, participants located their PE places (N = 2171) and reported transport mode (active/passive) used. Type of UZ (pedestrian/public transport/car) of participants’ home and PE places and distance from home to PE place were defined in GIS. For analyses, participants were grouped according to home UZ, shares of PE places in each UZ defined, and differences in total number of PE places and median distance tested with Kruskal-Wallis test. In each group, use of AT (vs. passive) to PE place was regressed for PE place’s UZ and distance, and adjusted for car availability, difficulty walking 2 km, age, sex, and years of education using a mixed model nested in participants.

**Results:**

In all groups, participants had more PE places in their home UZ than in other UZs. Median distance to and number of PE places did not differ across groups (for both p>.05). Overall, between-participant differences explained 22 % of total variance in AT. Compared to AT to PE place in home UZ, odds for AT were higher to PE place in car zones among those living in pedestrian (OR 5.1 95%CI 1.7-15.4) and public transport zones (OR 3.6 95%CI 1.3-8.4).

The odds for AT were lower to PE place in pedestrian zones among those living in public transport (OR 0.2 95%CI 0.1-0.4) or car zones (OR 0.02 95%CI 0.001-0.3). In all groups, longer distance was negatively associated with AT.

**Conclusions:**

Older adults’ transport mode choices cannot be concluded from the UZ of home or PE place. Proximity to PE places is important to facilitate AT use.

**Funding Source:**

Ministry of Education and Culture.

